# Targeted-Release Organic Acids and Essential Oils Improve Performance and Digestive Function in Broilers under a Necrotic Enteritis Challenge

**DOI:** 10.3390/ani10020259

**Published:** 2020-02-06

**Authors:** Nedra Abdelli, José Francisco Pérez, Ester Vilarrasa, Irene Cabeza Luna, Diego Melo-Duran, Matilde D’Angelo, David Solà-Oriol

**Affiliations:** 1 Animal Nutrition and Welfare Service (SNIBA), Department of Animal and Food Science, Universitat Autonòma de Barcelona, 08193 Bellaterra, Spain; nedra.abdelli@uab.cat (N.A.); josefrancisco.perez@uab.cat (J.F.P.); Diego.Melo@uab.cat (D.M.-D.); Matilde.Dangelo@uab.cat (M.D.); 2 FARMFAES-TECNOVIT, 43365 Alforja, Spain; ester.vilarrasa@gmail.com (E.V.); icabeza@farmfaes.com (I.C.L.)

**Keywords:** organic acids, aromatic compounds, microencapsulation, performance, intestinal histomorphology, microbiota, gut health, broiler

## Abstract

**Simple Summary:**

Controlling digestive diseases in the poultry industry is crucial to maximize profitability. Necrotic enteritis (NE) is a real threat for poultry that leads to high financial losses. Microencapsulated blends of organic acids and essential oils have gained increasing interest as feed additives that could alleviate the effects of these diseases by controlling the intestinal microbiota and enhancing the gut function of broiler chickens. Organic acids actually used as feed additives, including short-chain fatty acids (C1-C6), medium-chain fatty acids (C7-C12), and other organic acids, may show a range of variable physiological effects in the animals when combined with different phytogenic compounds. This study was designed to understand the mechanisms of action of these feed additives, their effect on intestinal morphology and growth performance, as well as their interaction with the gut microbiome. Our results provide evidence on the importance of designing proper combinations and doses of these additives to enhance growth performance, the microbiota profile, and histomorphology. Dietary supplementation of 0.5 g/kg of BUTYTEC-PLUS and 2 g/kg of ACITEC-MC as microencapsulated blends are recommended to improve broiler chickens performance under NE challenge due to their positive effect on gut microbiome and the absorptive capacity of the intestine.

**Abstract:**

An experiment was performed to evaluate the effect of four different microencapsulated blends of organic acids (OA) and nature-identical aromatic compounds (AC) on growth performance and gut health of broilers challenged with a recycled NE litter. A total of 600 one-day-old male Ross 308 broilers were randomly assigned to five treatments consisting of a basal diet (as negative control) supplemented with each of the tested microencapsulated blends: OA1 (malic and fumaric acid) + AC; 2.5 g/kg; OA2 (calcium butyrate+fumaric acid) + AC; 1.7 g/kg; MCFA (capric-caprylic; caproic and lauric acid) + AC; 2 g/kg; and MCFA + OA3 (calcium butyrate+fumaric and citric acid) + AC; 1.5 g/kg. The AC used was the same for all treatments; including cinnamaldehyde, carvacrol, and thymol (8:1:1), as major compounds. Three tested blends enhanced growth performance by improving intestinal histomorphology (*p* < 0.001). The tested blends enhanced the abundance of some beneficial families such as Ruminococcaceae and Lachnospiraceae; while reducing that of harmful ones such as Enterobacteriaceae and Helicobacteraceae. A further dose-response experiment showed that 0.5 g/kg of the blend 2 and 2 g/kg of the blend 4 improved growth performance and intestinal histomorphology of chickens on d 42 and decreased fecal Enterobacteriaceae and *C. perfringens* counts. Similar effects to the previous experiment were observed for cecum microbiota.

## 1. Introduction

With the pressure of increasing awareness and changing mindsets of the consumers, poultry production is currently facing an important challenge consisting of how to deal with serious issues related to digestive diseases to maintain gut health under the antibiotic free rearing program [1]. In this context, necrotic enteritis (NE) and Eimeria coccidiosis are considered the most important digestive infectious diseases in chickens. NE is a widespread disease commonly diagnosed in poultry flocks that is caused by the overgrowth of commensal *Clostridium perfringens*, a spore forming, gram-positive, anaerobic, rod-shaped bacterium [2]. Although the primary causative agent is *Clostridium perfringens* types A and C, several additional factors have been reported as predisposing factors, such as cereal type in the diet, dietary protein levels, anti-nutritional factors, coinfection with other pathogens (particularly coccidiosis), as well as environmental and management factors such as stress, high animal density, and immunosuppression [3].

Global economic losses associated with enteric diseases in the poultry industry are estimated at US$ 6 billion year [4] due to increased mortality in case of acute clinical NE, and reduced growth performance, greater medication costs, and elevated risk of contamination of poultry products in the case of subclinical NE. The latter is more prevalent where *Clostridium perfringens* toxins, such as NetB toxin, damage the structure and function of epithelial cells leading to gut inflammation [1] accompanied by a disruption of the gut microbial community, impair gut barrier function [5] and thus, infected birds exhibit increased gut permeability and depressed growth [6].

For decades, NE and coccidiosis have been kept under control using antimicrobials and ionophore coccidiostats [7]. Antimicrobial pre-mixes and preventive or metaphylactic uses in large group of pigs and poultry are the main characteristics of those countries where antimicrobial consumption remains high. However, oral formulations generally result in higher exposure of the gastrointestinal microbiome to the antimicrobials, which is of particular concern in terms of a potential source of resistant bacteria [8]. The concern over the evolution and spread of antimicrobial resistance, which represents a potential threat for human and animal health, has led to an increasing interest in animal production schemes based on low or free antibiotic exposure.

Short- and medium-chain fatty acids, as well as essential oils (EO), can be considered promising candidates for preventing NE. EO have been reported to possess in vitro antimicrobial and antioxidant activities against a wide range of pathogenic bacteria [9], as well as to improve intestinal integrity and fortify the mucosal barrier [10], and to enhance cellular and humoral immunity [11]. On the other hand, short-chain fatty acids (SCFAs) are either simple mono-carboxylic acids such as formic, acetic, propionic, and butyric acids or carboxylic acids with the hydroxyl group such as lactic, malic, tartaric, and citric acids or short-chain carboxylic acids containing double bonds like fumaric and sorbic acids [12]. Although dietary supplementation of SCFAs may modulate microbiota through their bactericidal and bacteriostatic activity [13], they have also been shown to stimulate the expression of genes regulating growth, division, differentiation, proliferation, and apoptosis of epithelial cells [14]. They have been shown to improve performance and modulate resistance of broilers to diseases [15]. Medium-chain fatty acids (MCFAs), including caproic, caprylic, or capric acid possess a strong antibacterial activity against various gram-negative bacteria like *E. coli* and *Salmonella* and gram-positive bacteria like *Enterococcus*, *Staphylococcus,* and *Clostridia* [16] through targeting the bacterial cell membrane and the various essential processes that occur within and at the membrane. Other processes such as cell lysis, inhibition of enzyme activity, or impaired nutrient uptake may also contribute to inhibition of bacterial growth [17].

Combining EO and organic acids has shown to be efficacious due to the reported synergism between both compounds [18]. In fact, EO may increase the permeability of cell membranes which allows organic acids to diffuse easily into the microbial cells. Recently, microencapsulation of organic acids and EO has shown to prevent their absorption in the upper part of digestive tract, while allowing a higher bioactivity towards the lower gastrointestinal tract [19].

In the present study, it is hypothesized that a microencapsulated botanical and acidifier active combination would prevent the performance decrease associated with NE by affecting the intestinal microbiota and digestive function in broiler chickens. Thus, the aim of the present study was to investigate the efficacy of different microencapsulated blends containing short and medium-chain fatty acids, calcium butyrate, and nature-identical aromatic compounds (thymol, cinnamaldehyde, and carvacrol as major compounds) on performance and gut health of broilers under challenging conditions of NE. 

Two experiments were carried out, in which the first trial aimed to determine the design of combinations with a high efficiency, while the second trial focused on finding the optimal dose for each combination.

## 2. Materials and Methods 

### 2.1. Ethics Statement

All animal experimentation procedures were approved by the animal Ethics Committee (CEEAH) of the Universitat Autònoma de Barcelona (number code: CEEAH 1043R2) and were performed in accordance with the European Union guidelines for the care and use of animals in research [20].

### 2.2. Birds and Experimental Design

#### 2.2.1. Trial 1

A total of 600 one-day-old Ross 308 male broiler chickens obtained from a local hatchery were used in a 41-day-experiment. The room was provided with 50 floor pens (4 lines of 15, 10, 10, and 15 pens, respectively, divided by 2 central feeding aisles). Upon arrival, chicks were weighed and randomly assigned according to initial body weight (BW) into 5 experimental groups, each with 10 replicates and 12 birds per replicate, and continuously controlled over a period of 41 days. A non-medicated (no antibiotic or anticoccidial drug) wheat–corn–soybean meal-based diet was used as the basal diet for all treatments. Dietary treatments were then produced by supplementing the basal diet with the tested feed additives: Organic acids (OA) plus nature identical aromatic compounds (AC). The mixture of aromatic compounds used was the same for all treatments and contained cinnamaldehyde, carvacrol, and thymol (8:1:1). These were obtained by synthesis or isolated through chemical processes, which are chemically identical to natural flavoring substances. The experimental treatments consisted of the basal diet as a negative control (NC) and 4 commercial microencapsulated products (Tecnovit, Alforja, Spain) as follows: **1.-** NC + OA1 (malic and fumaric acid) + AC, (ACITEC-A-GR, 2.5 g/kg); **2.-** NC + OA2 (cacium butyrate + fumaric acid) + AC, (BUTYTEC PLUS, 1.7 g/kg); **3.-** NC + MCFA (capric-caprylic acid, caproic acid, lauric acid) + AC, (ACITEC-M1, 2 g/kg); and **4.-** NC + OA3 (calcium butyrate + fumaric acid + citric acid) + MCFA + AC, (ACITEC-MC, 1.5 g/kg). Experimental doses of the commercial products were included as recommended by the company in commercial conditions.

#### 2.2.2. Trial 2

A total of 810 one-day-old Ross 308 male broiler chickens obtained from a commercial hatchery were used in a 42-day dose-response experiment. The room had 90 floor pens (2 lines of 23 and 2 lines of 22 pens each, divided by 2 central feeding aisles). Upon arrival, chicks were weighed and randomly assigned according to their initial BW into 10 experimental groups, each with 9 floor pens and 9 birds per replicate, and continuously controlled over a period of 42 days. The experimental treatments consisted of 5 increasing doses of two products selected from trial one: 0 g/kg for both products considered as negative control (NC; basal diet with recycled litter); basal diet supplemented with 0.5, 1, 2, and 4 g/kg of OA2 + AC or 1, 2, 4, and 8 g/kg of OA3 + MCFA + AC with recycled litter; and the positive control (PC) consisting of basal diet and non-contaminated new litter.

The chickens were weighed and feed disappearance was determined at 0, 10, 28, and 41 days of age (42 days for the second trial). Mortality rate and body weight of dead birds were also recorded daily. From these values, the average daily feed intake (ADFI), average daily gain (ADG), and feed conversion ratio (FCR) corrected by mortality were calculated.

### 2.3. Animal Husbandry

Both trials were carried out in a commercial growing poultry unit (Vila-rodona, Tarragona, Spain). Chicks were obtained from a commercial hatchery, where they were in ovo vaccinated according to the standard vaccination program, against Marek disease, Gumboro disease, and infectious bronchitis. Nonetheless, none of the chicks used in either trial received the coccidiosis vaccination. The birds were given 24 h of light for the first 2 days, which was reduced to 23 h of light and 1 h of dark from d 3 to d 10, and 18 h of light and 6 h of dark from d 11 until the end of the experimental period. The relative humidity was maintained between 50% and 70%.

### 2.4. Experimental Diets

The chickens were given a 3-phase feeding program in both experiments, consisting of a starter (0 to 10 d), grower (11 to 28 d), and finisher (29 to 41/42 d for first trial and second trial, respectively) diets. Table 1 lists the composition of the antibiotic-free and coccidiostat-free basal diet used during each phase. All diets were formulated to meet the requirements for maintenance and growth for broilers according to CVB poultry guidelines [21]. All chickens were given ad libitum access to feed in mash form and water. Housing facilities and birds were inspected twice daily (morning and afternoon) as regards general health status, feed and water availability, temperature, mortality, and any unexpected events. Samples were taken from all diets used, ground, and stored at 4 °C for their subsequent analysis in duplicate.

### 2.5. Necrotic Enteritis Challenge Procedure

The floor area of 1.125 m² (1.50 × 0.75 m) and 0.96 m² (1.20 × 0.80 m) per pen for the first and the second trial, respectively, was covered with 10% clean wood shavings and 90% recycled litter material. The recycled litter material was selected from a commercial poultry flock where it was claimed there was clinical NE, previously characterized for its content of mesophilic aerobic bacteria (>10^5^ CFU/g), Enterobacteriaceae (4.2 × 10^4^ CFU/g), filamentous fungi, yeasts, sulphite-reducing anaerobes (1.2 × 10^4^ CFU/g), and *Clostridium perfringens* (>10^5^ CFU/g). The challenging method consisting of exposing broilers to a contaminated litter characterized by high *Clostridium perfringens* counts, was previously used [22]. Moreover, wheat was included in the starter (15%), growing (20%), and finishing (25%) diets without xylanases, with the aim of increasing digesta viscosity and accentuating the dietary challenge.

### 2.6. Sampling Procedure and Analyses

#### 2.6.1. Feed

Diet proximate analyses were performed following AOAC methodology [23]: Dry matter (Method 934.01), crude protein (Method 968.06), crude fat (Method 2003.05), and crude fiber (Method 962.09). Gross energy was determined by an adiabatic calorimeter (IKA C-4000, Janke-Kunkel; Staufen, Germany).

#### 2.6.2. Bacteria Counts

For the second trial, 3 pooled fecal samples per treatment were collected from the negative control, the positive control, the lowest and the highest dose for each product for monitoring the evolution of Enterobacteriaceae, lactic bacteria, and *Clostridium perfringens* load using a quantitative count at d 14, 28, and 42.

From the faeces samples, a bank of decimal dilutions was prepared, in sterile Ringer’s solution, in order to proceed to the count and detection of: total Enterobacteriaceae, total lactic bacteria, and total *Clostrididum perfringens.*

The culture media used were: MacConkey agar in the case of Enterobacteriaceae, MRS agar for the count and detection of lactic bacteria, and TSN agar for *Clostridium perfringens* count. The incubation conditions were adequate for each determination, following the traditional methodologies in microbiology [24]. All trials were performed in triplicate.

#### 2.6.3. Acute Phase Proteins

At the end of each experiment, the bird with the closest BW to the mean of the pen was selected and blood samples were aseptically collected from the wing vein into vacutainers. Blood samples were centrifuged at 4000× *g* for 15 min to obtain the serum that was immediately stored at −20 °C for further analysis of acute phase proteins. 

The concentration of serum amyloid A (SAA) and alpha-1-acid glycoprotein (AGP) were determined using a solid phase sandwich ELISA (de Life Diagnostics, Knyperseley, UK) following the manufacturer’s recommendations.

Afterwards, the bird was killed by intracardiac administration of sodium pentobarbital (30 mg/kg BW), and jugular exsanguination for tissue sampling. The gastrointestinal tract was immediately dissected and content from ileum and ceca were collected for microbiome sequencing. Ileal tissue was collected in both trials to perform the histomorphological analysis.

#### 2.6.4. Histomorphological Analysis

At the midpoint between Meckel’s diverticulum and the ileo-cecal junction, ileal samples of about 5 cm were collected. For the first trial, tissue sections (5 μm) were fixed in 4% paraformaldehyde and then embedded in paraffin. Afterwards, the sections were prepared, and stained with hematoxylin-eosin. For the second experiment, the preparations were deparaffinized and hydrated before being subjected to PAS (Periodic acid-Schiff) staining with Schiff’s reagent for morphometric analyses and goblet cells count. For both trials, samples were analyzed using a light microscope. The morphometric variables measured included villus height, crypt depth, villus height to relative crypt depth ratio (V:C), and number of goblet cells and lymphocytes (only for the first trial). Ten villi were measured for each sample and only complete and vertically oriented villi were evaluated. The mean from 10 villi per sample was used as the mean value for further analysis.

#### 2.6.5. Preparation of the 16S rRNA Gene Amplicon Library for MiSeq Sequencing

The composition and structure of the sampled microbial communities was assessed through amplifying and sequencing the V3-V4 variable regions of the 16S rRNA gene. The Illumina Miseq sequencing 300 × 2 approach was used.

##### Library Preparation and Sequencing

Ileal and ceca contents (250 mg) for the first and the second trial, respectively, were collected from one bird per replicate for DNA isolation using the commercial MagMAX CORE Nucleic Acid Purification Kit 500RXN (Thermo Fisher, Barcelona, Spain), following the manufacturer’s instructions. Mock community DNA was included as control (Zymobiomics Microbial Community DNA). 

Samples were amplified using primers specific to the V3-V4 regions of the 16S rRNA DNA (V3-V4-Forward5′-TCGTCGGCAGCGTCAGATGTGTATAAGAGACAGCCTACGGGNGGCWGCAG-3′, V3-V4-Reverse 5′GTCTCGTGGGCTCGGAGATGTGTATAAGAGACAGGACTACHVGGGTATCTAATCC-3′) [25]. The library preparation was carried out in Microomics Systems S.L. (Barcelona, Spain).

##### Amplicon Sequences Processing and Analysis

Raw demultiplexed forward and reverse reads were processed using the following methods and pipelines as implemented in QIIME2 version 2019.4 with default parameters unless stated [26]. DADA2 was used for quality filtering, denoising, pair- end merging, and amplicon sequence variant calling (ASV, i.e., phylotypes) using *qiime dada2 denoise-paired* method [27]. Q20 was used as quality threshold to define read sizes for trimming before merging (parameters: --p-trunc-len-f and --p-trunc-len-r). Reads were truncated at the position when the 75th percentile Phred score felt below Q20 for for both forward and reverse reads. After quality filtering steps, average sample size of reads was determined and phylotypes were detected. ASVs were aligned using the *qiime alignment mafft method* [28]. The alignment was used to create a tree and to calculate phylogenetic relations between ASVs using qiime phylogeny fasttree method [29]. ASV tables were subsampled without replacement in order to even sample sizes for diversity analysis using *qiime diversity core-metrics-phylogenetic* pipeline. The sample with the smallest sample size was discarded in order to take advantage of the sequencing depth of the dataset. Subsequently, subsampling to the next lowest sample size was used for each comparison. Unweighted and weighted Unifrac distances were calculated to compare community structure [30]. Taxonomic assignment of ASVs was performed using a Bayesian Classifier trained with Silva V4 database (i.e., 99% OTUs database) using the *qiime feature-classifier classify-sklearn* method [31]. Unifrac distance matrices and ASV tables were used to calculate principal coordinates and construct ordination plots using R software package version 3.6.0

### 2.7. Statistical Analysis

For the first trial, statistical analyses were carried out on BW, ADG, ADFI, FCR, and histomorphological analysis with ANOVA using the GLM procedure of SAS software (SAS 9.4 Institute Inc., Cary, NC, USA). Normal distribution and homocedasticity of variances was checked previous to the analysis by using the Shapiro-wilk test and Levene’s test from UNIVARIATE and GLM procedures, respectively. All performance and histomorphological data were analyzed according to a randomized complete block design, considering treatment groups as the source of variation, and the number of pens (individual broiler chickens for the histomorphology) as the experimental unit. Means were compared using the multiple comparisons Tukey test, and deemed significant at *p* ≤ 0.05. A *p*-value situated between 0.05 and 0.10 was considered a trend towards significance.

For the second trial, the linear and quadratic contrasts were used to compare effects of increasing dietary supplementation of each tested blend. A further analysis of the following orthogonal contrasts was performed: Contrast C1, comparing the NC and the PC, contrast C2: Comparing the PC and the dose of 2 g/kg OA3 + MCFA + AC, and contrast C3: Comparing the PC and the dose of 0.5 g/kg OA2 + AC.

Biostatistical analysis for microbiota was performed in open source software RStudio v.3.5.1. Diversity was analysed at OTU level using vegan package [32]. Richness and alpha diversity were calculated with raw counts based on Simpson, Shannon, and Inverse-Simpson estimators. Beta diversity was evaluated by multivariate ANOVA. Finally, differential abundance analysis was performed with taxa relative abundances under a zero-inflated log normal mixture model and *p*-values were corrected by false-discovery rate (FDR) with a metagenomeseq package [33].

## 3. Results

### 3.1. First Trial

#### 3.1.1. Growth Performance

Mortality was 0.36% and was not related to any dietary treatment. Table 2 shows the growth performance of the birds that was lower than Ross 308 standards, and confirmed that the experimental challenge impaired the growth of the animals. Birds supplemented with blends containing malic and fumaric acid (OA1), calcium butyrate and fumaric acid (OA2), or capric-caprylic acid, caproic acid, lauric acid, calcium butyrate, fumaric acid, and citric acid (MCFA + OA3) showed higher BW at d 41 and higher ADG041 (*p* < 0.001) than those fed the negative control. Chickens supplemented with the blend OA2 + AC showed the highest overall ADFI (*p* = 0.02). All tested blends improved the FCR041 (*p* < 0.001). 

#### 3.1.2. Histomorphological Analysis

As shown in Table 3, the villus length to crypt depth V:C ratios were significantly higher (*p* < 0.001) in those birds on the OA1 + AC, OA2 + AC, and MCFA + OA3 + AC treatments compared to those birds of the negative control. All tested blends reduced crypt depth (*p* < 0.001). However, no treatment effect was observed on goblet cell and intraepithelial lymphocyte counts.

#### 3.1.3. Acute Phase Proteins

No treatment effect was observed on the serum concentration of alpha-1-acid glycoprotein (*p* = 0.97) or on the serum concentration of serum amyloid A (*p* = 0.56), with a mean of 0.348 mg/mL and 119.8 ng/mL, respectively (data not shown).

#### 3.1.4. Ileal Microbiota Analysis

##### Alpha and Beta Diversity

**Figure 1:**
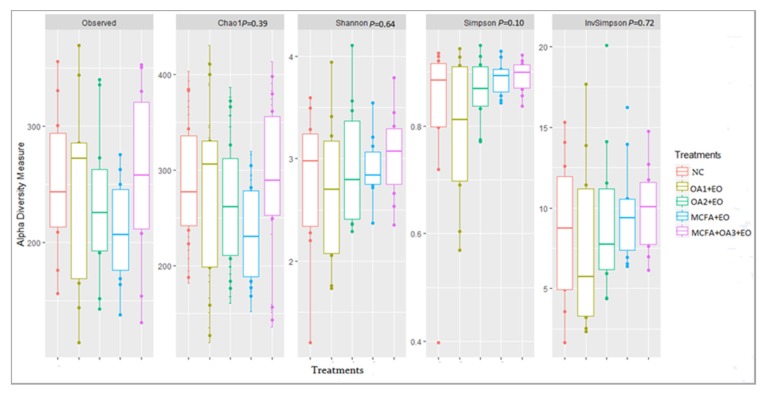
Alpha diversity indices of the ileal microbiota of broiler chickens at d 41 of age. (NC: Negative control; OA1: Malic acid + fumaric acid; OA2: Calcium butyrate + fumaric acid; OA3: Calcium butyrate + fumaric acid + citric acid + MCFA; MCFA: Capric-caprylic acid + caproic acid + lauric acid; AC: Cinnamaldehyde, carvacrol, and thymol as major compounds).

Alpha diversity indices showed a similar pattern distribution for all experimental treatments (within sample variability; Figure 1). None of the alpha diversity indices were statistically different among diets. The β-diversity was not affected by the experimental treatments (between sample variability; *p* = 0.50).

##### Composition of the Ileal Microbiota

**Figure 2:**
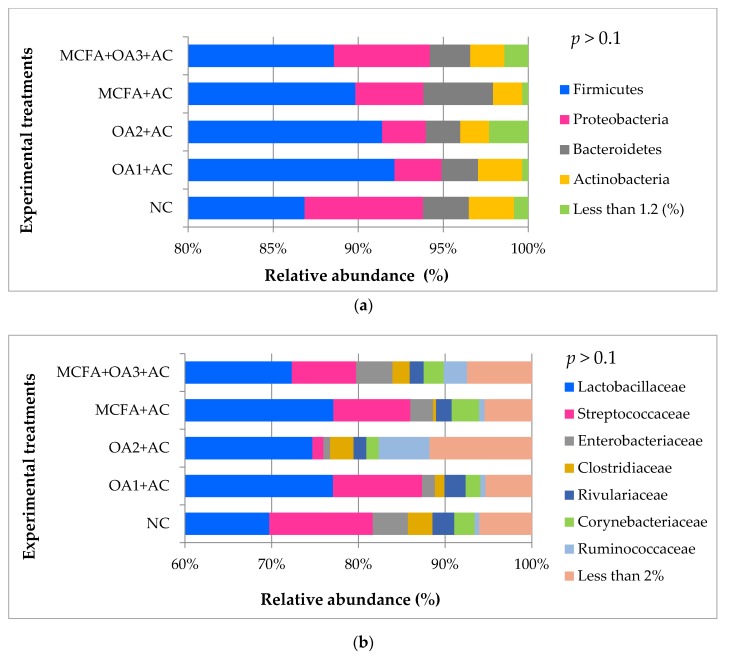

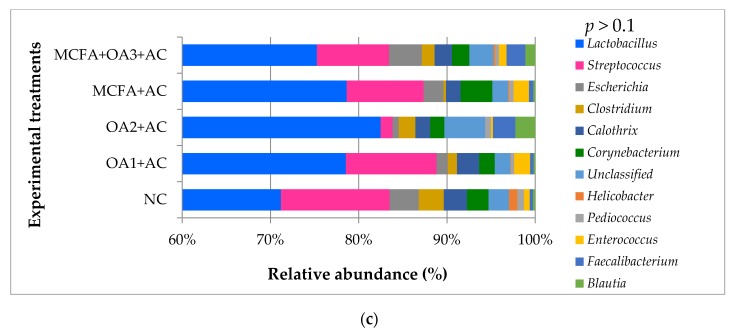
Relative abundance (%) of the main phyla (**a**), families (**b**), and genera (**c**) present in the ileal microbiota of broiler chickens at d 41 of age (Trial 1). NC: Negative control; OA1: Malic acid + fumaric acid; OA2: Calcium butyrate + fumaric acid; OA3: Calcium butyrate + fumaric acid + citric acid + MCFA; MCFA: Capric-caprylic acid + caproic acid + lauric acid; AC: Cinnamaldehyde, carvacrol, and thymol as major compounds).

No treatment effect was observed on ileal microbiota composition. The relative abundance of the main phyla, families and genera in the ileal microbiota of the birds is shown in Figure 2. Firmicutes, Bacteroidetes, Proteobacteria, and Actinobacteria were the most abundant phyla with no effect due to dietary treatments. The most abundant families were Lactobacillaceae, Streptococcaceae, and Enetrobacteriaceae. *Lactobacillus* and *Streptococcus* followed by *Escherichia* and *Clostridium* were the most frequent genera.

**Figure 3:**
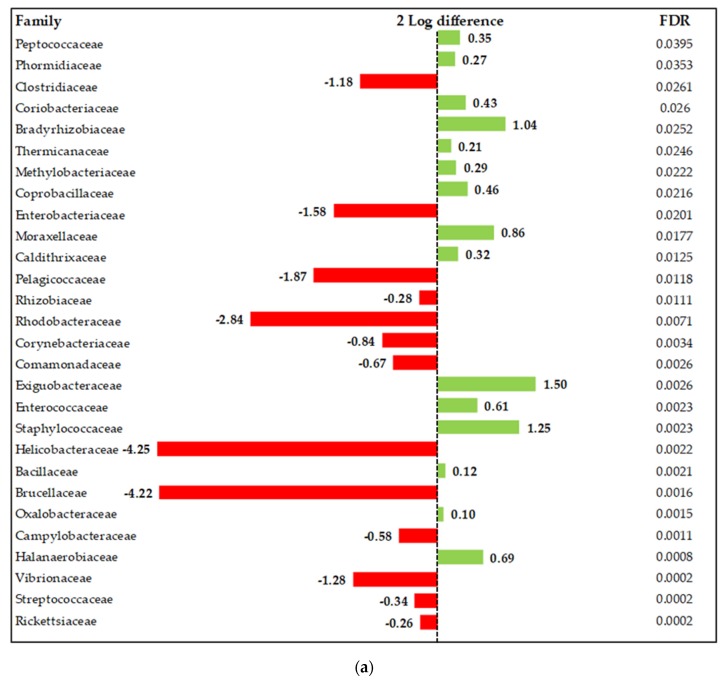

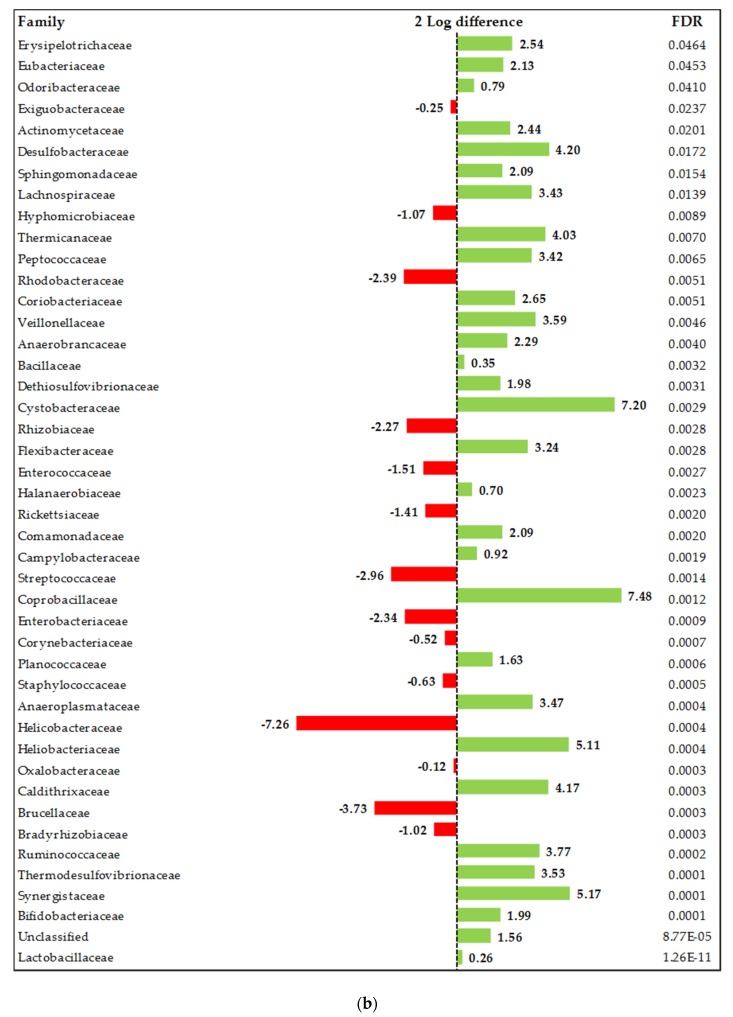

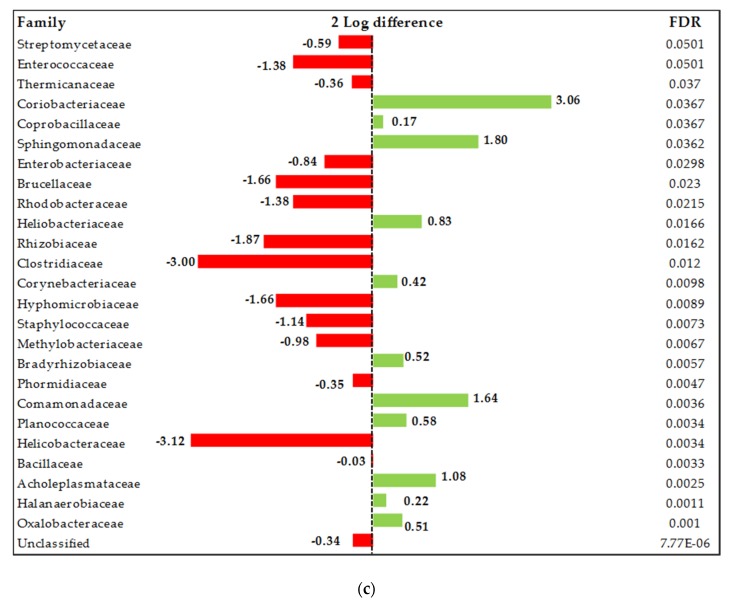

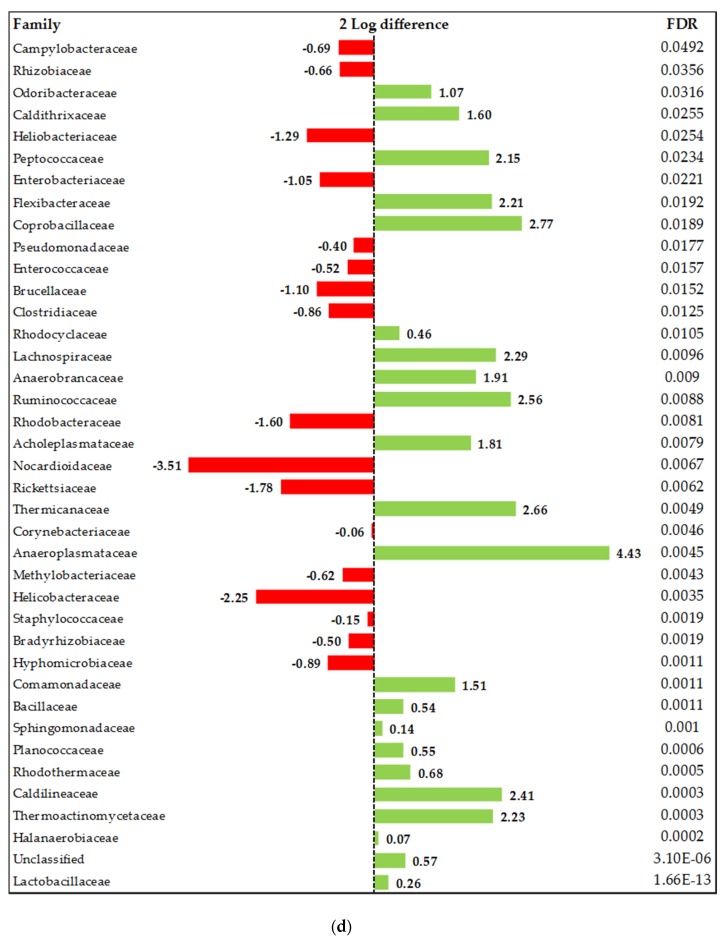
Differentially abundant taxa (family) from the ileum (in change and FDR-adjusted, *p* ≤ 0.05) on d 42 between OA1 + AC vs. NC (**a**); OA2 + AC vs. NC (**b**); MCFA + AC vs. NC (**c**); MCFA + OA3 + AC vs. NC (**d**). NC: Negative control; OA1: Malic acid + fumaric acid; OA2: Calcium butyrate + fumaric acid; OA3: Calcium butyrate + fumaric acid + citric acid + MCFA; MCFA: Capric-caprylic acid + caproic acid + lauric acid; AC: Cinnamaldehyde, carvacrol, and thymol as major compounds).

Nevertheless, although overall patterns were similar, a deeper examination of the individual metagenomics profiles was performed by means of log2 changes. Results showed some changes over 1-2 log2 individual taxa, when comparing all treatments containing microencapsulated blends of organic acids and AC with the negative control (Figure 3). Some families that contain relevant pathogenic taxa, such as Enterobacteriaceae, Helicobacteriaceae, Rickettsiaceae, and Clostridiaceae, decreased compared to the control treatment. Likewise, the abundance of some families shown to be beneficial for the host, such as Bifidobacteriaceae, Ruminococcaceae, and Lachnospiraceae, was enhanced in groups supplemented with the microencapsulated blends. 

### 3.2. Second Trial

#### 3.2.1. Growth Performance

The growth performance results are shown in Table 4. A linear-quadratic dose response analysis showed, for the overall parameters, a quadratic effect of OA2 + AC inclusion on BW, ADG, and ADFI (*p* < 0.001). These findings suggest that an improvement of growth performance could be observed up to a dose of 2 g/kg. However, higher doses may be associated with reduced growth performance. As for OA3 + MCFA + AC, a quadratic effect was observed only for BW42 (*p* = 0.02), while a linear response was observed for ADG042 (*p* = 0.004), ADFI042 (*p* = 0.05), and FCR042 (*p* = 0.01). Contrast C1 showed that chickens fed the PC had a higher BW42 (*p* = 0.01) as a result of better ADG042 (*p* = 0.03). Contrast C2 showed that supplementing 2 g/kg of OA3 + MCFA + AC improved BW10 (*p* = 0.03) and tended to improve FCR1028 (*p* = 0.08). The overall growth parameters showed no difference between PC and 2 g/kg of OA3 + MCFA + AC supplementation. Contrast C3 showed that the lowest dose (0.5 g/kg) of OA2 + AC improved the ADG042 (*p* = 0.05). No significant difference was observed for BW42 (*p* = 0.28), ADFI042 (*p* = 0.13), and FCR042 (*p* = 0.28).

**Figure 4:**
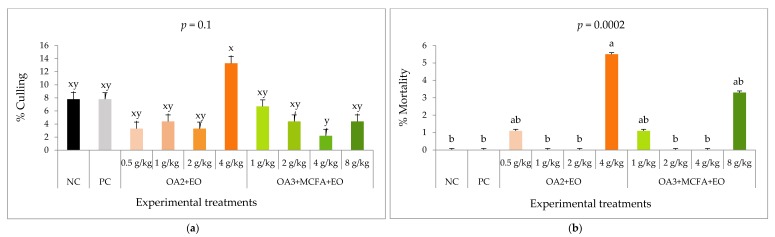
Culling (**a**) and mortality (**b**) calculated as a percentage from the total of chickens per treatment (90 chickens). ^a,b^ Means with different superscripts indicate significant differences (*p* ≤ 0.05). ^x,y^ Means with different superscripts indicate a tendency toward significance (*p* ≤ 0.1). NC: Negative control; PC: positive control; OA2: Calcium butyrate + fumaric acid; OA3: Calcium butyrate + fumaric acid+ citric acid + MCFA; MCFA: Capric-caprylic acid + caproic acid+lauric acid; AC: Cinnamaldehyde, carvacrol, and thymol as major compounds.

Culling and mortality rates are shown in Figure 4. Mortality was higher (*p* < 0.001), and culling rate tended to be higher (*p* = 0.10) with the highest dose of OA2+AC (4g/kg).

#### 3.2.2. Histomorphological Analysis

Results of the histomorphologiacal analysis of the ileum are shown in Table 5. A linear-quadratic dose response analysis showed a quadratic effect of OA2 + AC supplementation on CD and the ratio VH: CD (*p* < 0.001), suggesting an improvement of these two parameters up to a dose of 2 g/kg. However, a worsening could be observed when higher doses (4 g/kg) are used.

#### 3.2.3. Bacteria Counts

**Figure 5:**
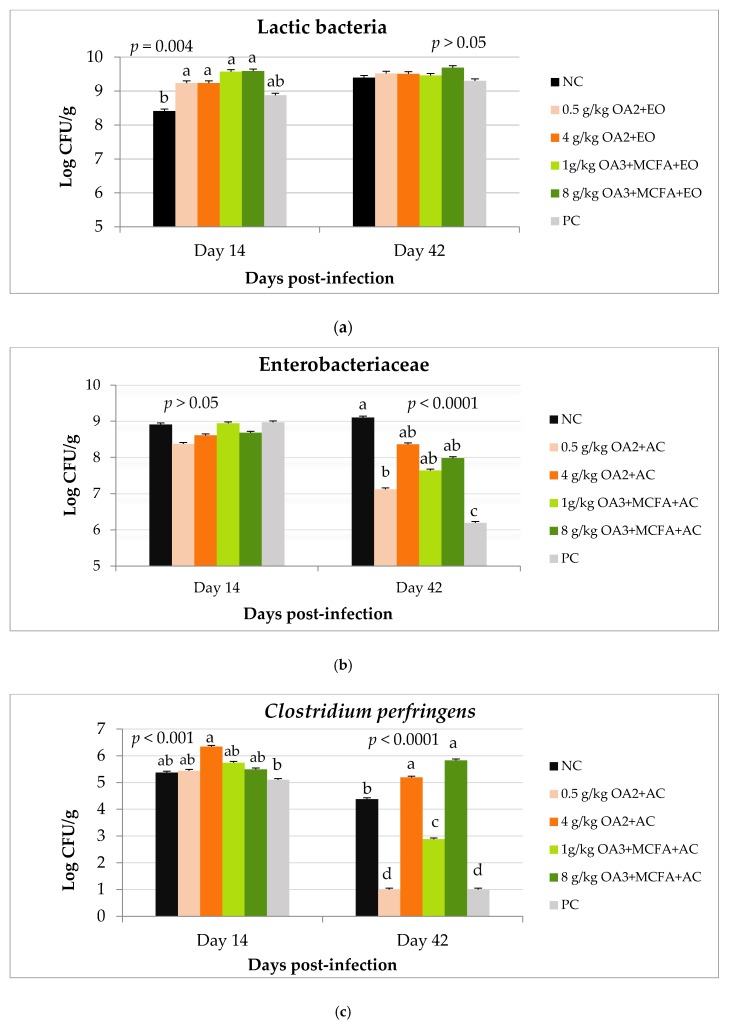
Effect of treatments on lactic bacteria (**a**), Enteribacteriaceae (**b**), and *C.perfringens* (**c**) count (log10 CFU) in feces collected on d 14 and 42 of age. OA2: Calcium butyrate + fumaric acid; OA3: Calcium butyrate + fumaric acid + citric acid + MCFA; MCFA: Capric-caprylic acid + caproic acid + lauric acid; AC: Cinnamaldehyde, carvacrol, and thymol as major compounds; NC: Negative control; PC: Positive control. ^a,b,c,d^ Means with different superscripts for the same day indicate significant difference between treatments (*p* ≤ 0.05).

The bacteria count results are shown in Figure 5. Baseline values were determined prior to the distribution of animals by collecting eight samples of feces from the transportation cages. With regards to Enterobacteriaceae, differences between dietary treatments were observed from d 28 of the experiment, where all doses of both blends showed reduced counts compared to the negative control (NC). On d 42, this effect remained only for the dose of 0.5 g/kg of OA2 + AC. A dietary treatment effect on *Clostridium perfringens* counts was observed on d 14, when the dose of 4 g/kg of OA2 + AC showed higher values compared to PC. This had been maintained on d 42. A similar effect was observed with the blend of OA3 + MCFA + AC where the dose of 8g/kg showed high count values on d 42. An effect of day-post-infection was observed for the doses of 1 g/kg of OA3 + MCFA + AC and 0.5 g/kg of OA2 + AC, where a significant decrease of *C. perfringens* count was observed at the end of the experiment. The supplementation of 0.5 g/kg of OA2 + AC resulted in count values similar to the PC.

#### 3.2.4. Cecal Microbiota Analysis

##### Alpha and Beta Diversity

The diversity of the cecal microbiota among dietary treatments was assessed using the α-diversity and β-diversity measurements. The challenge did not affect the α-diversity, as no difference was observed between the negative control and the positive one. However, the species richness of samples collected from chickens supplemented with both microencapsulated products was significantly higher than NC (Table 6).

**Figure 6:**
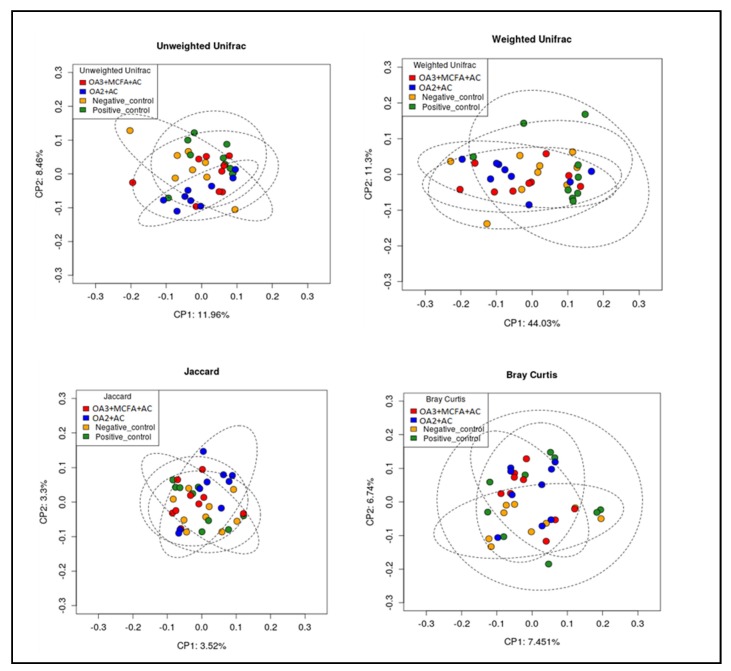
Effect of different dietary treatments on bacterial beta diversity on cecum of broilers on d 42 (Trial 2). (OA2: Calcium butyrate + fumaric acid; OA3: Calcium butyrate + fumaric acid + citric acid + MCFA; MCFA: Capric-caprylic acid + caproic acid + lauric acid; AC: Cinnamaldehyde, carvacrol, and thymol as major compounds).

No treatment effect was observed on β-diversity (between sample variability; *p* = 0.50, Figure 6).

##### Composition of the Cecal Microbiota

**Figure 7:**
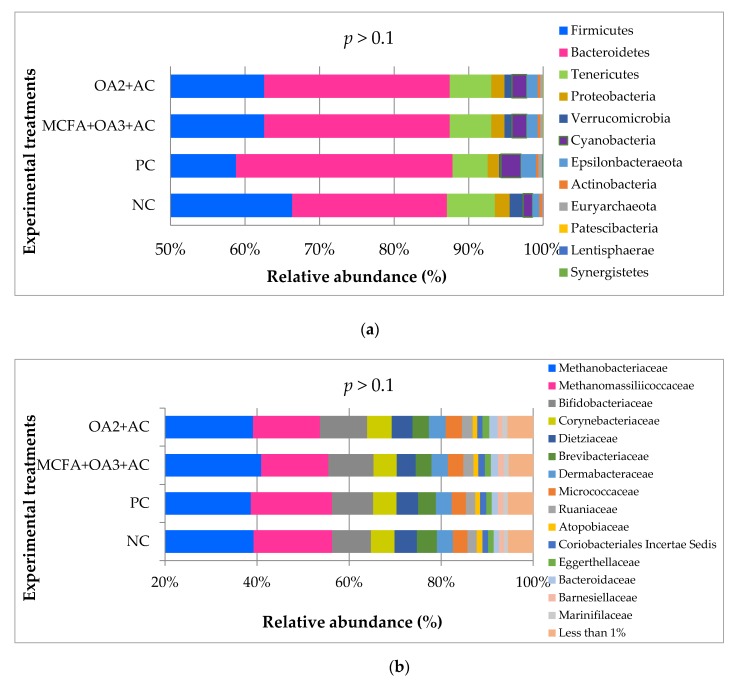

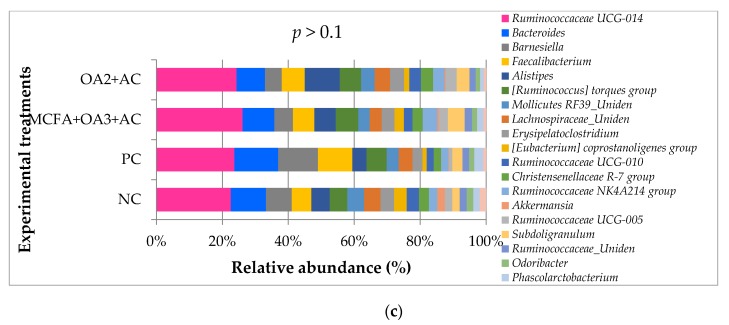
Relative abundance (%) of the main phyla (**a**), families (**b**), and genera (**c**) present in the cecum microbiota of broiler chickens at d42 of age (Trial 2). NC: Negative control; OA2: Calcium butyrate + fumaric acid; OA3: Calcium butyrate + fumaric acid + citric acid + MCFA; MCFA: Capric-caprylic acid + caproic acid + lauric acid; AC: Cinnamaldehyde, carvacrol, and thymol as major compounds; PC: Positive control.

**Figure 8:**
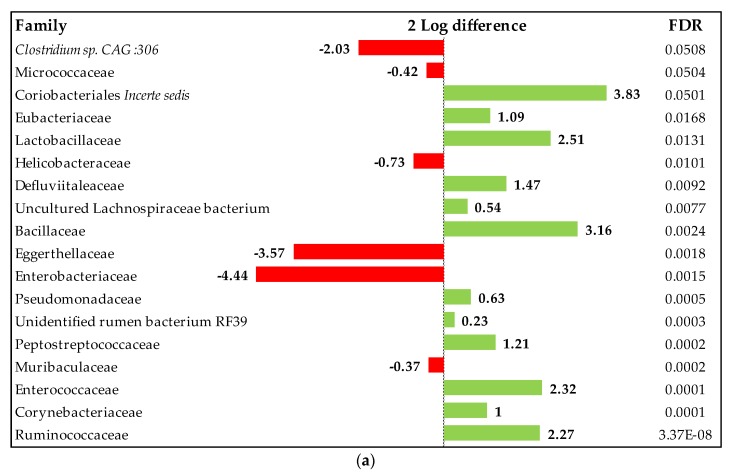

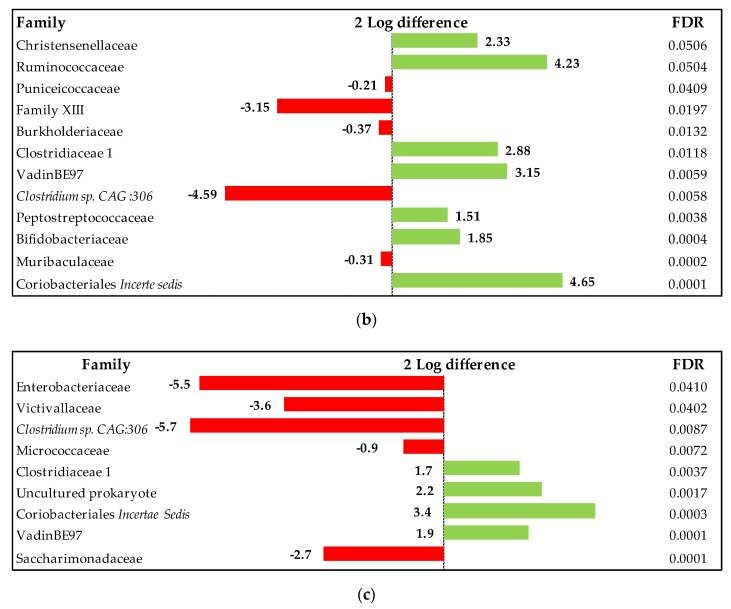
Differentially abundant taxa (family) from the cecum (in change and FDR-adjusted, *p* ≤ 0.05) on d 42 between OA3 + MCFA + AC vs. NC (**a**), OA2 + AC vs. NC (**b**), and NC vs. PC (**c**). OA2: Calcium butyrate + fumaric acid; OA3: Calcium butyrate + fumaric acid + citric acid + MCFA; MCFA: Capric-caprylic acid + caproic acid + lauric acid; AC: Cinnamaldehyde, carvacrol, and thymol as major compounds.

No treatment effect was observed on the cecum microbiota composition. The relative abundance of the main phyla, families, and genera in the ileal microbiota of the birds is shown in Figure 7. Firmicutes, Bacteroidetes, and Tenericutes were the most abundant phyla, without any effect due to dietary treatments. The most abundant families were Methanobacteriaceae, Methanomassiliicoccaceae, and Bifidobacteriaceae. *Ruminococcaceae UCG-014*, *Bacteroides* followed by *Barnesiella* and *Faecalibacterium* were the most frequent genera. Nevertheless, although overall patterns were similar, a deeper examination of the individual metagenomics profiles was performed by means of log2 changes (Figure 8). Results showed some changes over 1-2 log2 individual taxa when comparing OA3 + MCFA + AC treatment (2 g/kg) and OA2 + AC treatment (0.5 g/kg), to the negative control. Our results showed that the supplementation of OA3 + MCFA + AC increased Ruminococcaceae, Coriobacteriales *Incertae Sedis*, Eubacteriaceae, Lactobacillaceae, Bacillaceae, Corynebacteriaceae, and Peptostreptococcaceae, while reducing *Clostridium sp. CAG: 360*, Eggerthellaceae, and Enterobacteriaceae. OA2 + AC supplementation promoted Christensenellaceae, Ruminococcaceae, Peptostreptococcaceae, Vadin BE97, Clostridiaceae, Bifidobacteriaceae, and Coriobacteriales *Incerte Sedis*, while reducing *Clostridium sp. CAG: 360*, Clostridiales Family XIII, and Muribaculaceae.

## 4. Discussion

### 4.1. The Relevance of Organic Acids and Essential Oils Combination on Growth Performance

Induced *Clostridium perfringens* challenge did not result in clinical signs of NE and higher mortality of chickens, but it increased the morbidity that was evidenced by reducing growth performance up to 21% compared to the standard Ross 308 values. These results confirmed that the use of 90% recycled commercial litter contaminated with *Clostridium perfringens* combined with wheat inclusion without xylanases, is a suitable model to induce subclinical NE without promoting mortality of animals. In this scenario, experimental blends improved the growth of chickens compared to the negative control, except for the blend containing only MCFA and aromatic compounds (MCFA + AC). The highest BW gains were observed for birds fed the blend containing calcium butyrate and fumaric acid (OA2 + AC), followed by the blend containing the same compounds combined with citric acid and MCFA (OA3 + MCFA + AC). These findings suggested that embedding the active substances using a continuous film of vegetable fats provides better resistance to the acidic pH, allowing a slower release of these substances further down in the intestine, which resulted in improving growth performance. The performance responses could be related to an antimicrobial activity of organic acids inhibiting harmful microbiota and favoring the proliferation of beneficial bacteria [34]. Results regarding ileal and cecal microbiota showed a decrease of pathogenic taxa and an increase of some beneficial families in birds fed the microencapsulated blends compared to those receiving the negative control. These results were in line with those observed in feces where, for example, 0.5 g/kg of OA2 + AC significantly reduced *Clostridium perfringens* count on d 42 and Enterobacteriaceae from d 28. In fact, simple monocarboxylic acids such as formic, acetic, propionic, and butyric acids, or carboxylic acids bearing a hydroxyl group on the alpha carbon such as lactic, malic, and tartaric acids possess a strong antimicrobial activity [35]. Salts of some of these acids have been shown to enhance broilers performance, and short-chain carboxylic organic acids, such as sorbic and fumaric acids containing double bonds, also have antifungal activity [36]. The principal mode of action of organic acids is that non-dissociated forms can diffuse through lipophilic bacteria, mold membranes, disrupt the enzymatic reaction, and disorder transport systems of the bacteria [37]. Following the penetration of organic acids into bacterial cytoplasm, the non-ionized ones decompose to H (H+) ions and (A−) ions, resulting in a decline of the pH inside the bacteria. These changes are known to activate a specific mechanism (H^+^ - ATPase pump) that aims to return the intracellular levels to normal pH. The process requires energy, which would result in reduced energy availability for cell proliferation leading to a reduced bacterial growth [38]. Other effects related to a low internal pH are inhibition of glycolysis, prevention of active transport, and interference with signal transduction [39].

The effects of organic acids on the growth performance of broiler chickens could be also related to their ability to enhance protein digestion, influence intestinal cell morphology, stimulate pancreatic secretions, act as a substrate for the intermediary metabolism, improve retention of many nutrients (e.g., chelating minerals), increase intestinal integrity, and affect electrolyte balance in the feed and intestine [39,40]. Several authors also reported a beneficial effect of essential oils on feed digestion through increasing bile salt secretion and stimulating the enzymatic activities of intestinal mucosa and pancreas [41]. These beneficial effects of both organic acids and essential oils may be further potentiated when these are combined [42,43] and protected to avoid the active material to be metabolized and absorbed in the proximal part of the digestive tract [44]. In the present study, organic acids combined with AC affected the histomorphology and integrity of small intestine as showed by the villus height and crypt depth of OA1, OA2, or MCFA + OA3 groups. All tested blends reduced the crypt depth of the ileum that resulted in improved VH: CD ratio, which is an indicator of the absorptive capacity of the small intestine [45]. Several authors [44,46,47] pointed out their promoting effect on the development of gastrointestinal mucosa and villus height. In fact, enteric infection may damage the epithelium and compromise villus height leading to increase crypt depth indicating greater enterocyte-cell flow, and more steady cell-renewal rate within the digestive tract usually resulted from the increased sloughing [48]. These constant renewal processes demand more energy and protein, leading to diverting nutrients away from productive purposes. Our findings are in line with several studies [49,50]. However, numerical improvement [51] or no growth performance effects have been reported by other authors [52]. Disparity among studies could depend on the specific used organic acids and AC, their combination, diet formulation, doses, or differences in the underlying microbial challenge.

### 4.2. High Doses of the Additives May Become Deleterious

Our results showed a dose-dependent effect on productive performance and intestinal integrity where the best results were obtained with a dose of 2 g/kg for the blend of calcium butyrate, fumaric acid, citric acid, MCFA, and AC (OA3 + MCFA + AC) and 0.5 g/kg of the blend containing calcium butyrate, fumaric acid combined with AC (OA2 + AC). Higher dietary levels were associated to compromised productive performance resulting from shorter villus height, deeper crypts, and thus, reduced VH:CD ratio. High doses were also associated with higher culling and mortality rates, and higher fecal counts of Clostridium *perfringens*. It could be argued that a high dietary inclusion of organic acids may cause a damage [53], allowing more nutrients to drain from the mucosa into the lumen, which could favor the proliferation of intestinal *Clostridium perfringens* and cause more lesions. In the same sense, a study conducted by Timbermont et al. [54], showed that butyric acid (164.5 and 123 g/ton in starter and grower feed, respectively) combined with MCFA, mainly lauric acid (150 and 112.5 g/ton in starter and grower feed, respectively) and essential oils (thymol, cinnamaldehyde, and essential oil from eucalyptus; 90 and 67.5 g/ton in starter and grower feed, respectively) significantly reduced the number of birds with macroscopic lesions. However, the beneficial effect was lost when higher concentrations were used (330 and 250 g/ton of butyric acid combined with 360 and 270 g/ton of MCFA, and 240 and 180g/ton of essential oils for starter and grower feed, respectively). Several authors reported beneficial effect of low concentration of butyrate on promoting mucosal barrier function, while excessive butyrate disrupted it (100 mM and 8 mM of butyrate for Barcelo et al. [55]; Peng et al. [56], respectively).

### 4.3. The Relevance of Organic Acids and Essential Oils Combination on Ileum and Caeca Microbiota

The poultry gastrointestinal tract (GIT) is densely harbored by microorganisms, being in close and intensive interaction with the host and digesta particles. They are involved in the exchange of nutrients, and modulate the host gut morphology, physiology, and immunity [57]. The end-products of intestinal microbial fermentation are short-chain fatty acids (SCFAs), involved in the regulation of intestinal blood flow, intestinal immune responses, and mucin production as well as the stimulation of enterocyte growth and proliferation [58]. Among SCFAs, butyrate has gained a specific interest, being the main source of energy for epithelial cells and colonocytes. It also stimulates mucin synthesis and intestinal motility, cell proliferation and differentiation, while suppressing inflammatory diseases [59]. Thus, enhancing butyrate-producing bacteria would be of great interest for improving animal gut health and productivity. Unfortunately, we did not measure SCFAs in digesta and, consequently, we could not establish the correlations between these concentrations and relative abundance of bacterial taxa. However, as compared to the negative control, the tested microencapsulated blends increased the abundance of family members of Lachnospiraceae (*Coprococcus, Roseburia, Anaerostipes*) and Ruminococcaceae (*Faecalibacterium, Anaerotruncus*). These families, belonging to the Firmicutes phylum, express enzymes favoring the production of butyrate over propionate [60]. Supplementation of OA2 + AC also increased the abundance of Erysipelotrichaceae family, also known as Clostridium cluster XVI, that harbors different butyrate-producing bacteria [61,62]. It also increased the abundance of Bifidobacteriaceae that play an important role in pathogen exclusion and gut barrier maintenance due to their great production of SCFA through simple carbohydrates and oligosaccharides degradation [63]. Other studies reported that Actinobacteria, and mainly Bifidobacteria species, through inducing regulatory T-cells, can modulate the immune-inflammatory and autoimmune response [64,65]. Another family whose abundance was enhanced by OA2 + AC supplementation was Actinomycetaceae. Belonging to this family, *Streptomyces spp*, by producing Streptomycin, exerts a strong antimicrobial action against a number of gram-negative bacteria such as *Escherichia coli, Enterobacter, Salmonella*, and *Brucella* [66]. Moreover, ionophores, extensively used as anticoccidials, are fermentation products of Streptomyces and other fungi species. The abundance of Coriobacteriaceae that can produce high levels of SCFAs resulting in competitive exclusion of unfavorable microorganisms [67] was enhanced by the majority of tested blends. In addition, tested blends reduced Helicobacteraceae abundance by reducing *Helicobacter pullorum*, which has been also isolated from cecal epithelial cells [68] and shown to have a pathological outcome in the gut of chickens [69].

The first experiment of the current research showed that better performance responses were observed by the supplementation of OA2 + AC and OA3 + MCFA + AC through enhancing the abundance of beneficial families and reducing that of harmful ones in the ileum. A similar effect was observed in the cecum. The role of some families whose abundance changed in the cecum was previously discussed in the ileum microbiota.

Supplementation of OA2 + AC reduced Clostridiales Family XIII abundance that was previously found to be linked to broilers showing high FCR values [70] suggesting that their high abundance may result in compromised bird performance [71]. It also increased the abundance of Peptostreptococcaceae, normal commensal bacteria that have been shown to be higher in gut microbiota of healthy animals compared to those experiencing dysbiosis of the intestinal microbiota. This indicates that this family is involved in the maintenance of the gut homeostasis and enhancement of the barrier function [72].

## 5. Conclusions

In summary, dietary supplementation of microencapsulated blends of either BUTYTEC-PLUS or ACITEC-MC alleviates the negative impact of NE infection through modulating the gut microbiome. The positive effects on gut microbiome may enhance the absorptive capacity of the intestine by increasing the VH:CD ratio, which leads to improved productive performance. Results from the second trial show that inclusion doses up to 2 g/kg of BUTYTEC-PLUS and 8 g/kg of ACTEC-MC result in similar feed utilization efficiency, survival, and growth performance as the non-challenged positive control. However, these effects are dose-dependent as high inclusion rates such as 4 g/kg of BUTUYTEC-PLUS appear to promote detrimental effects on chickens. Consecuently, dietary supplementations of 0.5 g/kg of BUTYTEC-PLUS and 2 g/kg of ACITEC-MC are recommended to improve broiler chickens performance under NE challenge.

## Figures and Tables

**Table 1:** Ingredients and nutrient composition (% as fed-basis, unless otherwise indicated) of basal diet.

Items	Starter	Growing	Finishing
**Ingredient composition (%)**			
Maize	41.20	40.30	40.50
Wheat	15.00	20.00	25.00
Soybean meal 48	28.80	32.60	25.50
L-lysine HCL	0.15	0.07	0.05
DL-methionine	0.23	0.13	0.14
L-threonine	-	-	0.05
Soy oil	1.10	1.40	-
Palm oil	-	2.50	6.00
Extruded soybean meal	10.00	-	-
Limestone	1.09	0.70	0.70
Dicalcium phosphate	1.49	1.44	1.08
Salt	0.20	0.20	0.20
Vitamin-mineral premix *	0.40	0.40	0.40
Sodium bicarbonate	0.34	0.26	0.27
**Calculated composition (%)**			
Dry matter	88.1	88.1	88.4
G.E (kcal/kg)	3009	3101	3249
Crude protein	22.0	21.0	18.0
Lysine	1.35	1.18	1.06
Methionine	0.58	0.46	0.43
Ca	0.95	0.78	0.67
Total P	0.64	0.62	0.53
Available P	0.45	0.44	0.37
**Analyzed composition (%)**			
Dry matter	90.2	90.4	90.6
GE, kcal/kg	4081	4332	4395
Crude protein	22.5	21.3	18.5
Ether extract	4.8	6.0	7.9
Crude fiber	3.7	4.7	3.8

(-) Ingredient not incorporated. (*) Provided per kg of feed: Vitamin A (retinyl acetate) 10.000 IU; vitamin D3 (cholecalciferol) 4.800 IU; vitamin B1 (Thiamine) 3 mg; vitamin B2 (riboflavin) 9 mg; vitamin B3 (Nicotinamide) 51 mg; vitamin B6 (pyridoxine hydrochloride) 4.5 mg; vitamin B9 (folic acid) 1.8; vitamin B12 (cyanocobalamin) 0.04 mg; vitamin E (DL-α-Tocopheryl acetate): 45 mg; vitamin K3 (Menadione) 3 mg; pantothenic acid (calcium D-pantothenate) 16.5 mg, biotin (D-(+)-biotin) 0.15 mg; Chloride of choline 350 mg; iron (FeSO4) 54 mg; iodine (Ca(IO3)2) 1.2 mg; zinc (ZnO) 66 mg; manganese (MnO) 90 mg; copper (CuSO4) 12 mg; selenium (Na2SeO3) 0.2 mg; 6-Phytase EC 3.1.3.26: 1500 FYT; Butylated hydroxytoluene (BHT) 25 mg; Colloidal silica 45 mg, Sepiolite 1007 mg.

**Table 2:** Growth performance of chickens fed with experimental diets from d 1 to 41 (Trial 1).

Items	Experimental Treatments					SEM	*p*-Value
	NC	OA1 + AC	OA2 + AC	MCFA + AC	MCFA + OA3 + AC		
BW (g)							
d0	42.8	42.7	42.8	42.7	42.9	0.05	0.4008
d10	228.4 ^c^	240.5 ^a,b^	229.3 ^b,c^	242.6 ^a^	237.9 ^a,b,c^	2.84	0.0026
d28	1020 ^y^	1093 ^x^	1101 ^x^	1100 ^x^	1141 ^x^	28.8	0.0803
d41	1942 ^b^	2188 ^a^	2285 ^a^	2085 ^a,b^	2203 ^a^	51.3	0.0005
ADG (g/d)							
d0–10	18.6 ^c^	19.8 ^a,b^	18.6 ^b,c^	20.0 ^a^	19.5 ^a,b,c^	0.28	0.0022
d11–28	44.0	47.4	48.4	47.6	50.1	1.70	0.1116
d29–41	70.9	84.3 ^a,b^	91.0 ^a^	75.8 ^b,c^	81.7 ^a,b^	2.74	<0.0001
d0–41	46.3 ^b^	52.3 ^a^	54.6 ^a^	49.8 ^a,b^	52.7 ^a^	1.268	<0.0001
ADFI (g/d)							
d0–10	32.0 ^x^	31.1 ^x,y^	31.1 ^x,y^	30.5 ^y^	32.0 ^x,y^	0.41	0.0721
d11–28	77.5	82.8	84.0	80.5	82.9	1.78	0.1248
d29–41	162.3 ^a,b^	167.2 ^a,b^	173.9 ^a^	158.4 ^b,c^	170.1 ^a,b^	3.57	<0.0001
d0–41	88.5 ^b^	96.9 ^a,b^	99.6 ^a^	93.0 ^a,b^	98.1^a,b^	1.93	0.0248
FCR							
d0–10	1.72 ^a^	1.57 ^a,b^	1.67 ^a,b^	1.53 ^b^	1.64 ^a,b^	0.032	0.0051
d11–28	1.82	1.77	1.76	1.69	1.70	0.035	0.1203
d29–41	2.29 ^a^	1.98 ^b^	1.91 ^b^	2.01 ^a,b^	2.08 ^a,b^	0.042	0.0007
d0–41	1.87 ^a^	1.78 ^b^	1.78 ^b^	1.75 ^b^	1.79 ^b^	0.034	<0.0001

^a,b,c^ Values with different letters within a row indicate a significant difference at *p* ≤ 0.05. ^x,y^ Values with different letters within a row indicate a trend toward statistical significance at *p* ≤ 0.1. NC: negative control; OA1: malic acid + fumaric acid; OA2: calcium butyrate + fumaric acid; OA3: calcium butyrate + fumaric acid + citric acid + MCFA; MCFA: capric-caprylic acid + caproic acid + lauric acid; AC: cinnamaldehyde, carvacrol and thymol as major compounds.

**Table 3:** Effect of experimental treatments on histomorphology of ileum of broiler chicken at d 41 (Trial 1).

Items	Experimental Treatments					Statistics	
	NC	OA1 + AC	OA2 + AC	MCFA + AC	MCFA + OA3 + AC	SEM	*p*-Value
Villus height (μm)	828.5 ^c^	1044.0 ^a,b^	1088.4 ^a^	925.1 ^b,c^	1054.6 ^a,b^	35.90	<0.0001
Crypt depth (μm)	219.6 ^a^	174.5 ^b^	179.2 ^b^	191.5 ^b^	182.5 ^b^	4.90	<0.0001
VH:CD ratio	3.79 ^c^	6.04 ^a^	6.10^a^	4.89 ^b^	5.82 ^a^	0.22	<0.0001
Goblet cells density/100 μm of villus height	15.8	13.1	11.4	13.5	13.9	1.07	0.6134
Intraepithelial lymphocyte density/100 μm of villus height	7.9	6.6	6.0	7.1	6.8	0.37	0.6424

^a,b,c^ Values with different letters within a row indicate a significant difference at *p* ≤ 0.05. NC: Negative control; OA1: Malic acid + fumaric acid; OA2: calcium butyrate + fumaric acid; OA3: Calcium butyrate + fumaric acid + citric acid + MCFA; MCFA: Capric-caprylic acid + caproic acid + lauric acid; AC: Cinnamaldehyde, carvacrol, and thymol as major compounds.

**Table 4:** Effect of dietary treatments on productive performance of chickens during the different phases and the entire experimental period (Trial 2).

Items	Experimental Treatments										*p*-Value			
	NC	OA2 + AC (A)				OA3 + MCFA + AC (B)				SEM				
		0.5 g/kg	1 g/kg	2 g/kg	4 g/kg	1 g/kg	2 g/kg	4 g/kg	8 g/kg		Linear		Quadratic	
											A	B	A	B
BW (g)														
d0	39.3	39.4	39.4	39.4	39.4	39.4	39.4	39.4	39.4	0.04	0.85	0.85	0.22	0.4
d10	228.5	246.3	234.6	234.3	227.2	240.5	249	243.2	239.8	5.01	0.15	0.42	0.23	0.03
d28	1132	1283	1255	1241	1203	1194	1296	1263	1247	23.1	0.78	0.01	0.001	0.001
d42	2369	2598	2561	2573	2327	2469	2549	2549	2527	42.8	0.007	0.06	<0.0001	0.02
ADG (g/d)														
d0–10	18.9	20.7	19.5	19.5	18.8	20.1	21.0	20.4	20.0	0.46	0.13	0.34	0.41	0.03
d0–42	55.5	60.9	60.0	60.3	54.5	57.8	59.8	59.8	59.2	1.07	0.006	0.004	<0.0001	0.11
ADFI (g/d)														
d0–10	26.8	27.5	26.2	27.8	26.9	26.7	28.9	27.9	27.6	0.78	0.88	0.56	0.66	0.17
d0–42	95.1	102.6	101.4	100.6	95.2	98.3	102.1	99.9	101.1	1.46	0.08	0.05	0.0002	0.1
FCR														
d0–10	1.42	1.33	1.34	1.43	1.43	1.33	1.38	1.37	1.38	0.042	0.1	0.29	0.47	0.73
d0–42	1.71	1.68	1.69	1.67	1.75	1.70	1.71	1.68	1.71	0.018	0.13	0.01	0.42	0.96

NC: Negative control; OA2: Calcium butyrate + fumaric acid; OA3: Calcium butyrate + fumaric acid + citric acid + MCFA; MCFA: Capric-caprylic acid + caproic acid + lauric acid; AC: Cinnamaldehyde, carvacrol, and thymol as major compounds.

**Table 5:** Effect of dietary treatments on histology of the ileum at the end of the experiment (Trial 2).

Items	Experimental Treatments										*p*-Value			
	NC	OA2 + AC (A)				OA3 + MCFA + AC (B)				SEM				
		0.5 g/kg	1 g/kg	2 g/kg	4 g/kg	1 g/kg	2 g/kg	4 g/kg	8 g/kg		Linear		Quadratic	
											A	B	A	B
Villus height, VH (μm)	803.7	872.1	895.0	873.7	773.5	909.9	847.4	843.6	850.8	36.15	0.20	0.92	0.29	0.56
Crypt depth, CD(μm)	160.9	149.5	148.8	146.0	181.9	148.1	147.1	158.2	152.2	6.28	0.002	0.85	<0.0001	0.69
Ratio VH:CD	5.0	5.8	6.1	6.0	4.3	6.2	5.8	5.4	5.7	0.27	0.002	0.81	<0.0001	0.46
Goblet cells Density/100 μm of villus height	21.7	22.2	21.1	20.8	29.1	19.5	19.3	22.6	23.2	1.53	0.003	0.17	0.05	0.55

NC: Negative control; OA2: Calcium butyrate + fumaric acid; OA3: Calcium butyrate + fumaric acid + citric acid + MCFA; MCFA: Capric-caprylic acid + caproic acid + lauric acid; AC: Cinnamaldehyde, carvacrol, and thymol as major compounds.

**Table 6:** Effect of dietary treatments on alpha diversity.

Index	NC	PC	OA2 + AC	OA3 + MCFA + AC	SEM	*p*-Value
Shannon	0.91 ^b^	0.93 ^a,b^	0.94 ^a^	0.95 ^a^	0.006	0.009
Simpson	3.01 ^b^	3.14 ^a,b^	3.31 ^a^	3.36 ^a^	0.074	0.008
Invsimpson	12.43 ^b^	15.60 ^a,b^	19.26 ^a^	19.82 ^a^	1.502	0.004

^a,b^ Values with different letters within a row indicate a significant difference at *p* ≤ 0.05. NC: Negative control; OA2: Calcium butyrate + fumaric acid; OA3: Calcium butyrate + fumaric acid + citric acid + MCFA; MCFA: Capric-caprylic acid + caproic acid + lauric acid; AC: Cinnamaldehyde, carvacrol, and thymol as major compounds; PC: Positive control.
